# Clinical Examination in the Diagnosis of Anterior Cruciate Ligament Injury: A Blinded, Cross-sectional Evaluation

**DOI:** 10.5435/JAAOSGlobal-D-22-00123

**Published:** 2023-02-08

**Authors:** Robert L. Kulwin, Gregory J. Schmidt, Dayton A. Snyder, Robert G. Klitzman

**Affiliations:** From the The Christ Hospital Network, Cincinnati, OH, (Dr. Kulwin), and Department of Orthopedic Surgery, Indiana University, Indianapolis, IN, (Dr. Schmidt, Dr. Snyder, and Dr. Klitzman).

## Abstract

This study was conducted to compare the effectiveness of clinical tests for anterior cruciate ligament (ACL) injury.

**Methods:**

This study prospectively evaluated the effectiveness of the Lachman test, anterior drawer test, and lever test in diagnosing ACL injury in 133 patients with knee pathology. The examiner was blinded to the patient's history, symptoms, and laterality of the pain at the time of examination. One hundred twenty-three patients in the study underwent MRI, and 90 went on to arthroscopy. The performance of the examination maneuvers and MRI was calculated.

**Results:**

This study showed notable differences in sensitivity and specificity between the Lachman test and the lever test and in specificity between the anterior drawer test and the lever test. The Lachman test was also found to be more sensitive than the anterior drawer. All ACL tears diagnosed by a composite of the physical examination maneuvers were confirmed by MRI. MRI findings were concordant with arthroscopic findings in all cases.

**Conclusions:**

The Lachman test and the anterior draw test demonstrated clinical utility, but the results of the lever test should be interpreted with caution. Clinical examination was found to be highly specific but less sensitive than MRI.

The physical examination remains a mainstay of orthopaedic diagnosis, and multiple physical examination maneuvers can be used to evaluate a possible anterior cruciate ligament (ACL) injury. Historically, the most commonly used tests are the Lachman test, the anterior drawer test, and the pivot shift test.^1,2^ A meta-analysis comparing these tests demonstrated that the Lachman test is the most valid test to evaluate ACL tears with a pooled sensitivity of 85% (95% confidence interval [CI], 83 to 87) and a pooled specificity of 94% (95% CI, 92 to 95). The anterior drawer test showed a sensitivity of 92% (95% CI, 88 to 95) and specificity of 91% (95% CI, 87 to 94) when tested in chronic rather than acute injuries. The pivot shift test has been shown to be very specific 98% (95% CI, 96 to 99) but has a low sensitivity of 24% (95% CI, 21 to 27).^3^ In addition, it can be difficult to perform in a clinic setting because of patient discomfort.^3^ The lever test, a more recent addition to the clinical diagnostic toolbox, has also been described with early results suggestive that it may be more useful than the Lachman test.^4-6^

However, the body of literature supporting the lever test is somewhat limited in comparison with the other clinical tests. In addition, although multiple studies have evaluated the utility of MRI in the evaluation of ACL injury, these studies were underpowered, retrospective, unblinded, and the strength of magnet and quality of image were not reported.^7-9^ As such, the exact role of not only the clinical tests, particularly the lever test, and advanced imaging in the initial evaluation of ACL injury remains unclear.^10^

The goal of this study was to better describe the statistical and practical utility of clinical tests in the evaluation of ACL injuries through a prospective, blinded, consecutive enrollment study with inclusion of the lever test.

## Methods

After Internal Review Board approval, patients were consecutively enrolled from December 1, 2017 through June 30, 2019. All patients referred for knee pathology were screened, and informed consent was obtained before enrollment in the study. The patient's chief report was blinded in the schedule as “knee pain.” While the patient was roomed by the clinic staff, the study was discussed, and informed consent was obtained if the patient was interested in being included in the study. Before the formal clinical visit, both knees were examined by the senior author (RGK) who was blinded to all patient information, history, and prior imaging including the laterality of the injury. Before examination, each of the six possible orders of the studied maneuvers was entered into the Choose for Me—Random Choice Maker smartphone application developed by Golden Key to prevent the results from any one examination affecting the interpretation of subsequent maneuvers. The lever test was done as described by Lelli et al^5^ in which the patient is positioned supine with the examiners fist under the calf and the other hand applying a downward force on the distal third of the quadriceps. A lever test was determined to be negative, or indicative of intact ACL, if the patient's heel rose off the examination table with the downward force on the quadriceps, whereas it was determined to be positive, or indicative of ACL tear, if the heel remained on the table after force application. The anterior drawer and Lachman tests were both done with a standard technique. The result for each examination was marked as positive or negative. After the examination was completed, the final diagnosis based on the examination findings was documented. The presence of effusion on examination was also noted. All MRIs were done on 1.5 T or 3.0 T scanners and were read by a musculoskeletal radiologist. No arthroscopic surgeries were done for diagnostic purposes only.

**Figure 1:**
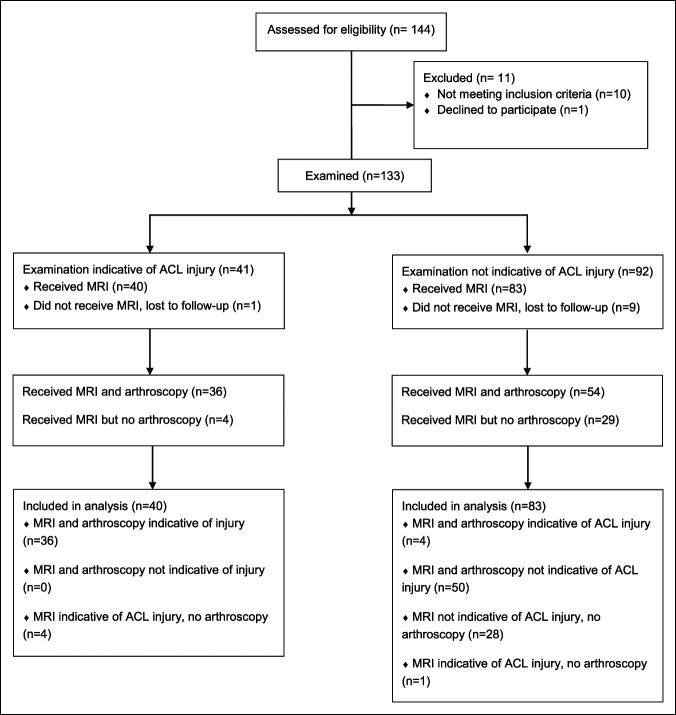
Consolidated Standards of Reporting Trials flow diagram demonstrating the progression of patients through the study. ACL = anterior cruciate ligament, MRI = magnetic resonance imaging

A total of 144 patients were assessed for eligibility. Inclusion criteria for the study were skeletal maturity, a chief report of acute knee pain, and a clinical indication for MRI or arthroscopy after evaluation. Exclusion criteria included: history of connective tissue disease or confounding preexisting conditions which alter the expected native anatomy of the lower extremity. One patient was excluded because they did not wish to participate, one patient was excluded because of history of tibial hemimelia, and nine patients were excluded because neither MRI nor arthroscopy was clinically indicated. A total of 133 patients were examined for this study. During the period of the study, 123 patients underwent MRI and 90 patients underwent arthroscopy (Figure 1). Descriptors of the study population are given in Table 1. The decision to proceed with arthroscopy was based on individual patient's presentation, imaging findings, and a discussion of the risks and benefits of surgery with the patient.

**Table 1:** Patient Characteristics

Age (yr)	Median	30
	Minimum	14
	Maximum	59
Sex	Female	65 (48.9%)
	Male	68 (51.1%)
Laterality of ACL tear	Left	66 (49.6%)
	Right	65 (48.9%)
	Bilateral	2 (1.5%)

ACL = anterior cruciate ligament

MRI was used as the benchmark in the evaluation of examination maneuvers, while arthroscopy was used as the benchmark in the evaluation of MRI. Sensitivity and specificity comparisons were done using the McNemar tests. Positive predictive value (PPV) and negative predictive value (NPV) comparisons were done using generalized score tests.^11^ Evaluation of the effect of effusion on the performance of the individual examination maneuvers was also completed using the Fisher exact test. Analyses were done using the R package DTComPair. Pre hoc power calculations were not feasible because the prevalence of ACL injury was unknown. Post hoc power calculations were based on the observed prevalence of an ACL injury at 37% (MRI) or 44% (arthroscopy) in this study. Assuming total discordance of 20% between the diagnostic tests, this study is powered to detect differences of 12% or more in sensitivity and specificity between diagnostic tests. For all analyses, statistical significance was set at *P* < 0.05.

## Results

A total of 133 patients were examined for this study. During the period of the study, 123 patients underwent MRI and 90 patients underwent arthroscopy (Figure 1). Descriptors of the study population are given in Table 1. Of the 123 injured knees, 40 were diagnosed with ACL tear on clinical examination and 45 on MRI findings. Ninety patients underwent arthroscopy, with 40 diagnostic for ACL tear. Of note, five of the patients with MRI findings of ACL tear did not undergo arthroscopy. All patients with MRI indicative of ACL tear who underwent arthroscopy had intraoperative evidence of ACL tear, and no patients were arthroscopically diagnosed with ACL tear without having MRI evidence of ACL tear. Thus, MRI was found to be 100% sensitive and specific in this study.

Of the 36 patients with a positive examination finding for ACL tear that elected for surgical management, 100% of the patients had this diagnosis confirmed and were treated with ACL reconstruction (ACLR). There were four patients who were not diagnosed with ACL tear after examination with positive MRI findings for partial ACL tear that subsequently underwent arthroscopy, while the fifth patient with discordant examination and MRI findings elected for nonsurgical treatment. Arthroscopic evaluation of these patients was indicative of ACL injury in all. Of those patients, two elected not to undergo ACLR because of lack of clinical symptoms of instability and two underwent ACLR.

Using MRI as the benchmark (Table 2), the lever test was found to be statistically inferior to the Lachman test in sensitivity, specificity, PPV, and NPV (*P* ≤ 0.001, 0.011, 0.002, and <0.001, respectively). It was also inferior to the anterior drawer test in specificity, PPV, and NPV (*P* = 0.011, 0.003, 0.022, respectively). While the specificities of the anterior drawer and Lachman were not significantly different, the anterior drawer was found to significantly less sensitive than the Lachman (*P* = 0.001). Compared with MRI, the Lachman test had inferior sensitivity (*P* = 0.046) with no significant difference in specificity (*P* = 0.317).

**Table 2:** Evaluation of the Maneuvers in Comparison With MRI as the Benchmark

Factor	Sensitivity (95% CI)	Specificity (95% CI)	Positive Predictive Value (95% CI)	Negative Predictive Value (95% CI)
Lachman	0.889 (0.765-0.952)	0.987 (0.931-0.999)	0.976 (0.874-0.999)	0.939 (0.865-0.974)
Anterior drawer	0.622 (0.476-0.749)	0.987 (0.931-0.999)	0.966 (0.828-0.998)	0.819 (0.729-0.884)
Lever	0.444 (0.309-0.588)	0.883 (0.793-0.937)	0.690 (0.508-0.827)	0.731 (0.633-0.811)

CI = confidence interval

The performance of the clinical examination as a composite of the three examination maneuvers is presented in Table 3. Overall, the composite examination showed a specificity of 1 and sensitivity of 0.89. The examination was not found to be significantly different from the Lachman in sensitivity (*P* = 1) or specificity (*P* = 0.317). Similarly, the examination was not significantly different from anterior drawer with specificity (*P* = 0.317). However, it was found to be significantly different from anterior drawer with sensitivity (*P* = 0.001). In addition, the performance of the composite examination was significantly different from the lever test in both sensitivity (*P* < 0.001) and specificity (*P* = 0.003).

**Table 3:** Comparison of Physical Examination Results With Physician's Diagnosis and MRI Findings on Anterior Cruciate Ligament Injuries

Physical Examination					Physician's Diagnosis		MRI Findings	
					ACL Torn	ACL Intact	ACL Torn	ACL Intact
	Lachman	Anterior Drawer	Lever	No. of Patients	N (%)	N (%)	N (%)	N (%)
Triple-positive	+	+	+	14	14 (100%)	0 (0%)	14 (100%)	0 (0%)
Double-positive	+	+	−	14	14 (100%)	0 (0%)	14 (100%)	0 (0%)
	+	−	+	3	3 (100%)	0 (0%)	3 (100%)	0 (0%)
	−	+	+	0	N/A	N/A	N/A	N/A
Single-positive	+	−	−	10	9 (90%)	1 (10%)	9 (90%)	1 (10%)
	−	+	−	1	0 (0%)	1 (100%)	0 (0%)	1 (100%)
	−	−	+	12	0 (0%)	12 (100%)	3 (25%)	9 (75%)
Triple-negative	−	−	−	69	0 (0%)	69 (100%)	2 (3%)	67 (97%)
Total				123	40 (33%)	83 (67%)	45 (37%)	78 (63%)

ACL, anterior cruciate ligament

The effect of an effusion at the time of examination on each examination maneuver was also evaluated (Table 4). Effusion was found to have a significant effect on the performance of both the anterior drawer (Relative Risk [RR] = 2.65) and lever (RR = 3.39) tests in increasing the risk of falsely negative examination findings, contributing to the decreased sensitivity of the test in comparison with the Lachman test.

**Table 4:** Effect of Effusion on Anterior Cruciate Ligament Test Accuracy

Factor	Lachman				Anterior drawer				Lever			
	True-positive	False-positive	Relative risk	*P*	True-positive	False-positive	Relative risk	*P*	True-positive	False-positive	Relative risk	*P*
Effusion	25	0	1.07	0.39	19	1	0.95	>0.99	12	4	1.22	0.69
No	15	1			9	0			8	5		

Factor	False-negative	True-negative	Relative risk	*P*	False-negative	True-negative	Relative risk	*P*	False-negative	True-negative	Relative risk	*P*
Effusion	3	20	3.85	0.13	9	19	2.65	0.04^a^	16	16	3.39	0.0005^b^
No	2	57			8	58			9	52		

ACL, anterior cruciate ligament; Fisher exact test.

a *P* < 0.05.

b *P* < 0.001.

## Discussion

This study evaluated the performance of physical examination maneuvers in the diagnosis of ACL injury. It indicates that the Lachman test and anterior drawer are both specific for ACL tear with a notable difference as compared with the Lever test. The Lachman test was found to be more sensitive than the anterior drawer and Lever tests, and these findings supported previously reported values for the sensitivity and specificity of the Lachman.^3^ However, while the anterior drawer was found to be less sensitive than the Lachman, it was more specific. Importantly, the findings of this study are contrary to the findings of the initial study by Lelli et al,^5^ which demonstrated 100% successful diagnosis of ACL tears and subsequent studies supporting the use of the lever test.^4,5,12,13^ As the lever test was inferior to the Lachman in both sensitivity and specificity and less specific than anterior drawer, the evidence presented here calls into question the diagnostic utility of the lever test.

Another finding of this study was the somewhat limited utility of MRI in the diagnosis of ACL injury in the setting of a positive clinical examination. No patients who were diagnosed with an ACL tear on clinical examination were found to have an intact ACL through advanced imaging or arthroscopy. However, it is important to note that secondary injury at the time of ACL tear is common, and MRI may still have utility in the evaluation of patients with ACL injuries to further assess for meniscal pathology or chondral injury.^14^ Although this study did not aim to evaluate the performance of MRI when evaluating for chondral damage or meniscal injury, difficulty in assessing for possible meniscus tears on MRI in the context of an ACL tear has been previously described.^15-17^

MRI can provide benefit in the setting of a negative examination after knee injury with concern for ACL tears. In this series, MRI diagnosed four ACL tears that were not diagnosed on examination and were confirmed arthroscopically. Of those four tears, two of the patients decided to not undergo ACLR due to the lack of clinical knee laxity.

There are several weaknesses in this study, first being that it is relatively underpowered. In addition, the performance of all clinical examinations by a senior attending surgeon may limit the generalizability of the study. It is a reasonable assumption that less experienced examiners may be less accurate in the evaluation of these injuries. Finally, the examiner had no clinical information on the patient, including which knee was prompting the visit for evaluation, but the presence of an effusion could not be blinded. Despite that, in clinical practice, knee effusion as well as patient history, mechanism, and symptoms would be expected to help rather than hinder a diagnosis of ACL tear.

The physical examination has long been the mainstay of orthopaedic evaluation and diagnosis. The Lachman and anterior drawer tests are both clinically useful and highly specific, although the anterior drawer is less sensitive than Lachman. However, the lever test should have a more limited role, and findings should be interpreted with caution. Although MRI is certainly a useful imaging modality in the evaluation of knee pathology and can provide insight into concurrent knee pathology, it may not always be necessary to confirm a diagnosis of ACL injury. However, the MRI may continue to offer clinical utility in the evaluation of secondary injuries in the setting of ACL tears. Additional studies with multiple examiners and a larger sample size are recommended to improve the generalizability of this study.
